# Novel United Kingdom prognostic model for 30-day mortality following transcatheter aortic valve implantation

**DOI:** 10.1136/heartjnl-2017-312489

**Published:** 2017-12-07

**Authors:** Glen P Martin, Matthew Sperrin, Peter F Ludman, Mark A de Belder, Simon R Redwood, Jonathan N Townend, Mark Gunning, Neil E Moat, Adrian P Banning, Iain Buchan, Mamas A Mamas

**Affiliations:** 1 Faculty of Biology, Medicine and Health, Farr Institute, University of Manchester, Manchester Academic Health Science Centre, Manchester, UK; 2 Cardiology Department, Queen Elizabeth Hospital, Birmingham, UK; 3 Cardiology Department, James Cook University Hospital, Middlesbrough, UK; 4 Cardiology Department, Guys and St Thomas' NHS Foundation Trust, London, UK; 5 Keele Cardiovascular Research Group, Keele University, Stoke-on-Trent, UK; 6 Cardiology Department, Royal Brompton and Harefield National Health Service (NHS) Foundation Trust, London, UK; 7 Cardiology Department, John Radcliffe Hospital, Oxford, UK

**Keywords:** aortic stenosis, transcatheter valve interventions

## Abstract

**Objective:**

Existing clinical prediction models (CPM) for short-term mortality after transcatheter aortic valve implantation (TAVI) have limited applicability in the UK due to moderate predictive performance and inconsistent recording practices across registries. The aim of this study was to derive a UK-TAVI CPM to predict 30-day mortality risk for benchmarking purposes.

**Methods:**

A two-step modelling strategy was undertaken: first, data from the UK-TAVI Registry between 2009 and 2014 were used to develop a multivariable logistic regression CPM using backwards stepwise regression. Second, model-updating techniques were applied using the 2013–2014 data, thereby leveraging new approaches to include frailty and to ensure the model was reflective of contemporary practice. Internal validation was performed by bootstrapping to estimate in-sample optimism-corrected performance.

**Results:**

Between 2009 and 2014, up to 6339 patients were included across 34 centres in the UK-TAVI Registry (mean age, 81.3; 2927 female (46.2%)). The observed 30-day mortality rate was 5.14%. The final UK-TAVI CPM included 15 risk factors, which included two variables associated with frailty. After correction for in-sample optimism, the model was well calibrated, with a calibration intercept of 0.02 (95% CI −0.17 to 0.20) and calibration slope of 0.79 (95% CI 0.55 to 1.03). The area under the receiver operating characteristic curve, after adjustment for in-sample optimism, was 0.66.

**Conclusion:**

The UK-TAVI CPM demonstrated strong calibration and moderate discrimination in UK-TAVI patients. This model shows potential for benchmarking, but even the inclusion of frailty did not overcome the need for more wide-ranging data and other outcomes might usefully be explored.

## Introduction

Transcatheter aortic valve implantation (TAVI) has emerged as the indicated treatment option for patients with aortic stenosis who are intermediate to high-risk surgical candidates.^1–3^ Assessment of procedural risk is predominantly undertaken by the Heart Team, guided by multiple clinical prediction models (CPM).^4^ To this end, CPMs such as the EuroSCORE^5^ or the Society of Thoracic Surgeons (STS) mortality score^6^ have been used to guide estimation of procedural risk and employed in randomised controlled trials of TAVI.^1–3^ However, surgical-based models are known to perform poorly at predicting mortality in TAVI patients.^7 8^

Consequently, there have been recent attempts to derive procedure-specific CPMs using national registries; examples include the FRANCE-2 model,^9^ the Italian OBSERVANT model^10^ and the American College of Cardiology (ACC) model.^11^ Primarily, one is interested in using such TAVI CPMs to underpin procedure audit analyses across TAVI centres, to facilitate discussion of risk with the patient and to aid comparison of randomised trials. However, existing models have only moderate performance when applied in samples outside of their development cohorts.^12^ Indeed, within the UK, there is no validated CPM for predicting mortality post-TAVI,^13^ and heterogeneity in the variables recorded among national registries restricts the application of existing models into UK practice. As such, developing a CPM for UK-TAVI patients is vital, especially since one of the fundamental incentives for collecting national registry data is to monitor centre-level performance for audit and feedback purposes.

Therefore, the aim of this study was to develop and internally validate a multivariable TAVI CPM for predicting 30-day mortality in UK-TAVI patients (hereto called the UK-TAVI CPM), to facilitate clinical discussions around procedure risk with the patient during the consent process, and for national benchmarking/audit analyses.

## Methods

The reporting of this manuscript adheres to the TRIPOD checklist for the reporting of multivariable prediction models.^14^ The completed checklist is provided as online supplementary material.

10.1136/heartjnl-2017-312489.supp1 Supplementary file 1

### UK-TAVI Registry

The UK-TAVI Registry prospectively collects data for every TAVI procedure conducted in the UK (34 centres) through a web-based interface.^15^ This study included data on all TAVI procedures between January 2009 and December 2014. Procedures were predominately undertaken via transfemoral access, using either the Edwards SAPIEN or the Medtronic CoreValve families of devices (with their various iterations over time), although other access routes and valve types were available. The registry records information on patient baseline demographics, risk factors for intervention and procedural details. Frailty information was only recorded within the registry from January 2013 through the KATZ Index of activities of daily living,^16^ the Canadian Study of Health and Aging (CSHA) frailty scale^17^ and an indicator of poor mobility (as defined in the EuroSCORE II model^5^).

### Outcome and cohort definition

All-cause mortality tracking was independently obtained from the Office for National Statistics, with administrative censoring occurring on 31 May 2015; this information was used to define the binary endpoint of 30-day mortality after TAVI. Mortality information was unavailable for patients in Northern Ireland and Scotland; consequently, these patients were excluded, along with any patient in England and Wales with missing mortality information. Thus, the main development sample included all TAVI patients with 30-day mortality indication across England and Wales, between January 2009 and December 2014.

### Predictors

For development of the UK-TAVI CPM, we considered all variables available prior to the TAVI procedure (see online supplementary table 1 for the full list of considered covariates, with translation to the UK-TAVI Registry). Our decision to include access route as a candidate covariate was made in line with previous TAVI CPM development data sets,^9 11^ and represents the potential risk/complications/comorbidities associated with a non-transfemoral approach. Age and sex were always included in the model, with age being mean centred. Quadratic and cubic transformations of continuous variables were considered.

### Missing data

Multiple imputation by chain equations was used to handle missing data, with this analysis generating 10 imputed data sets.^18^ The imputation models for each baseline covariate included the majority of other variables in the UK-TAVI Registry and the survival data (both the survival event indicator and the cumulative baseline hazard^19^), the latter of which was included because this was used to derive 30-day mortality indication. Prior to analysis, we checked all the imputations to ensure convergence and checked that the distributions of observed and imputed values were similar. Since multiple imputation assumes a missing at random mechanism, we undertook a sensitivity analysis of this assumption as described below.

### Statistical analysis

**Figure 1:**
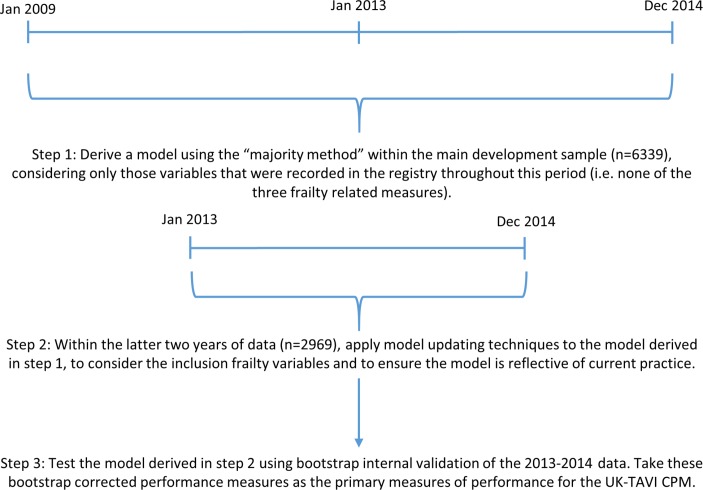
Flow chart illustrating the steps undertaken within the two-stage modelling strategy to derive and internally validate the UK-TAVI CPM. CPM, clinical prediction model; TAVI, transcatheter aortic valve implantation.

A two-stage modelling strategy was implemented to derive the CPM (figure 1). We first derived a logistic regression model within the 2009–2014 data, considering only those variables that were observed throughout this period (ie, none of the frailty measures), using the ‘majority method’ of developing CPMs within multiple imputed data.^20^ Here, the following steps were undertaken: (1) perform backwards selection using Akaike information criterion in each of the 10 imputed data sets, resulting in 10 (potentially unique) sets of predictors; (2) extract variables that were selected in >50% of the imputed data sets; (3) given the extracted variables, fit a model in each imputed data set, then pool the estimated coefficients and SEs across imputations according to Rubin’s rules.^18^ The Akaike information criterion for backwards selection corresponds to a P value of 0.157 for a variable with 1 df.

In the second stage, we updated this derived model using model-updating techniques within the last 2 years of data^21 22^ (figure 1). Here, a logistic regression model was fit to 30-day mortality within the 2013–2014 data, with the calculated linear predictor (LP) from the 2009–2014 derived model, KATZ, CSHA and poor mobility as covariates (see online supplementary methods for mathematical details). The likelihood ratio test was used to determine if any of the three frailty measures significantly improved the fit of the model, with only those meeting this criterion included in the final model. The reasoning for this second modelling step was twofold. First, it uses all available data as the foundations of the UK-TAVI CPM, while ensuring that the model is reflective of most contemporary practice/technology. Second, it provides a way of considering frailty variables within the model, which we hypothesised a priori would be important predictors of mortality,^23^ but were only recorded within the registry from January 2013.

Predictive performance was assessed through calibration and discrimination. Calibration is the agreement between the observed and expected risk, which was quantified by the calibration intercept and calibration slope, with reference values of 0 and 1, respectively.^24^ Discrimination of a model is its ability to distinguish those who experienced the outcome from those who did not, and was estimated with the area under the receiver operating characteristic (ROC) curve (AUC). Throughout, we use the term ‘apparent performance’ to denote the predictive performance of a model within the data set in which it was developed. As such, the apparent performance was obtained by applying the final model coefficients to each patient within the 2013–2014 data across the 10 imputed data sets and pooling performance results across imputations (using Rubin’s rules^18^). Internal validation was undertaken using bootstrap resampling of the 2013–2014 data to estimate optimism-corrected performance (figure 1)^22 25^; details of this process are given in the online supplementary material. In short, the bootstrap resampling represents sampling from the underlying population to estimate in-sample optimism, which can be subtracted from the apparent model performance. These internal validation results should be treated as the primary measures of predictive performance.

R V.3.4.0^26^ was used for all statistical analyses. Multiple imputation of the data set was completed using the mice package,^27^ and the package pROC was used for constructing ROC curves.^28^ All other codes were written by the authors and are available upon request.

### Sensitivity analyses

Instead of using multiple imputation, the first sensitivity analysis (singularly) imputed all missing continuous variables using the sex-matched median of the observed values, and missing categorical variables using the mode. For the majority of categorical variables this will result in a ‘risk factor absent’ assumption and corresponds to a viable missing not-at random assumption. Similar modelling steps to the main analysis were then performed using this single imputation strategy. The second sensitivity analysis performed the ‘majority method’ of variable selection directly within the 2013–2014 data set to examine the robustness of the two-stage modelling strategy.

## Results

**Figure 2:**
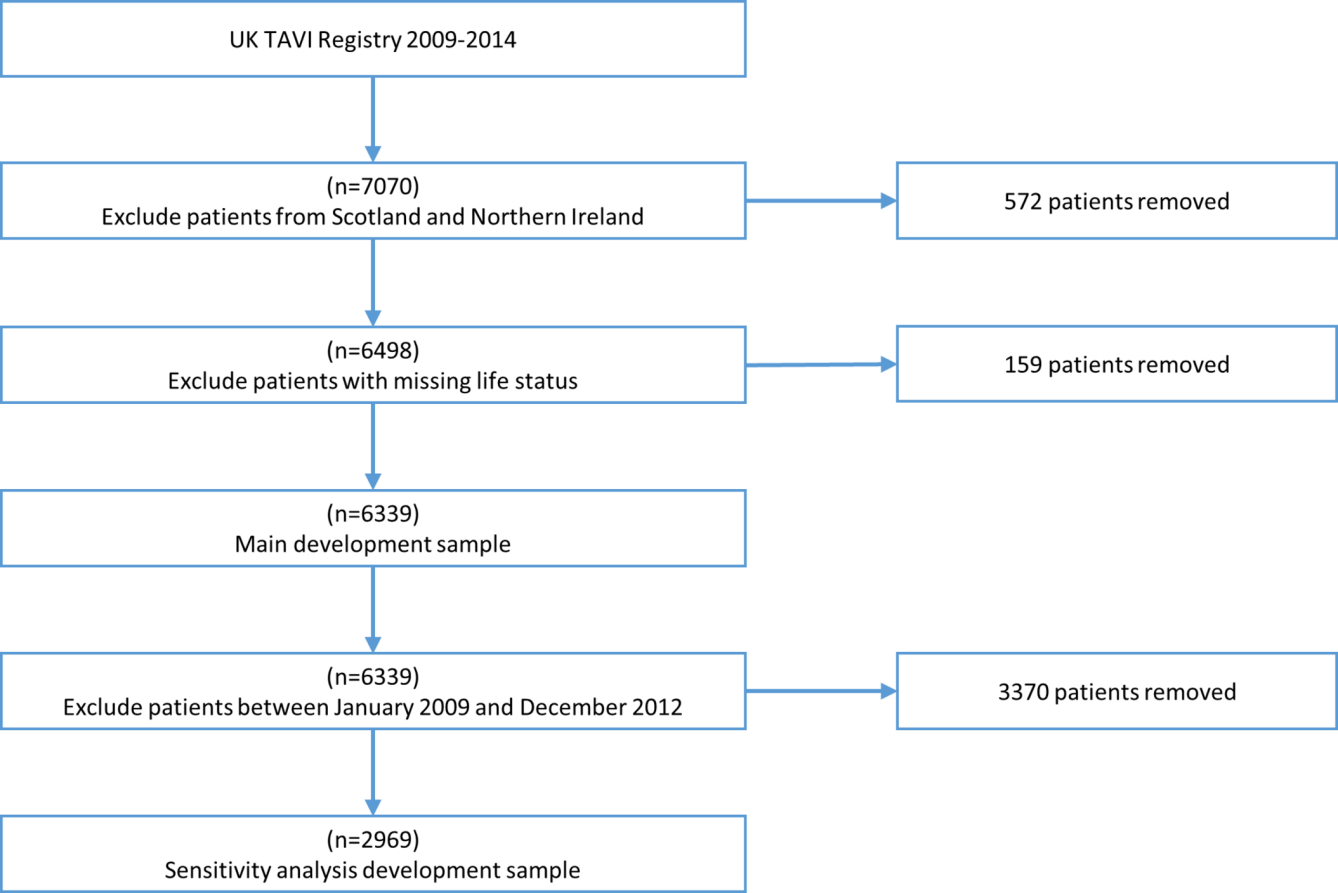
Patient flow chart through the exclusion criteria for both the main development sample and the sensitivity analysis that modelled using 2013/2014 data only. TAVI, transcatheter aortic valve implantation.

Between January 2009 and December 2014, up to 7070 patients were recorded in the registry. After excluding patients in Northern Ireland (n=379) and Scotland (n=193), a further 159 patients were removed due to missing life status. Hence, the development sample for the main analysis included 6339 patients (figure 2). Baseline characteristics and proportions of missing data for the main development cohort are given in table 1. The mean age of patients was 81.3 years, with 46.2% female. Most procedures were performed electively (12.5% non-elective), via transfemoral access (25.4% non-transfemoral access), and used a SAPIEN valve (56%). The proportion of missing data was low for most variables. The high percentages of missing data for the three frailty measures are due to these variables only being recorded from January 2013, after which the proportion of missing data for poor mobility, CSHA and KATZ was 1.79%, 1.65% and 11.0%, respectively.

**Table 1:** Baseline and procedural characteristics for patients in the main development cohort

Variable*	Summary (n=6339)	Missing (% of 6339)
Age, mean (range)	81.25 (29–101)	0 (0)
Female, n (%)	2927 (46.17)	22 (0.35)
Non-Caucasian, n (%)	249 (3.93)	65 (1.03)
Diabetic, n (%)	1463 (23.08)	35 (0.55)
Current or ex-smoker, n (%)	3240 (51.11)	245 (3.86)
Height (m), mean (range)	1.65 (1.10–2.36)	156 (2.46)
Weight (kg), mean (range)	74.23 (32–190)	129 (2.04)
Creatinine, µmol/L, mean (range)	114.2 (29–1044)	71 (1.12)
Dialysis, n (%)	122 (1.92)	66 (1.04)
MI within 30 days of TAVI, n (%)	60 (0.95)	33 (0.52)
Pulmonary disease, n (%)	1777 (28.03)	87 (1.37)
Cerebrovascular disease, n (%)	913 (14.40)	34 (0.54)
Extracardiac arteriopathy, n (%)	1462 (23.06)	84 (1.33)
Calcified aorta, n (%)	1076 (16.97)	73 (1.15)
Sinus rhythm, n (%)	4054 (63.95)	106 (1.67)
Previous cardiac surgery, n (%)	1990 (31.39)	35 (0.55)
Prior BAV, n (%)	694 (10.95)	32 (0.50)
Previous PCI, n (%)	1272 (20.07)	34 (0.54)
Critical preoperative state, n (%)	98 (1.55)	81 (1.28)
NYHA class IV, n (%)	1089 (17.18)	42 (0.66)
Poor mobility, n (%)	662 (10.44)	3423 (54.00)
CSHA frail, n (%)	1165 (18.38)	3419 (53.94)
KATZ<6, n (%)	851 (13.42)	3696 (58.31)
PA systolic >60 mm Hg, n (%)	740 (11.67)	1816 (28.65)
Aortic peak gradient, mean (range)	74.56 (3.35–200)	259 (4.09)
Aortic valve area, mean (range)	0.68 (0.2–2)	388 (6.12)
LVEF<50%, n (%)	2421 (38.19)	55 (0.87)
LMS*>*50%, n (%)	275 (4.34)	138 (2.18)
Non-elective procedure, n (%)	790 (12.46)	7 (0.11)
Non-transfemoral access, n (%)	1607 (25.35)	13 (0.21)
Valve type		29 (0.46)
Edwards SAPIEN valve, n (%)	3553 (56.05)	
Medtronic CoreValve, n (%)	2531 (39.93)	
Other, n (%)	226 (3.57)	

*Variable definitions are given in online supplementary table 1.

BAV, balloon aortic valvuloplasty; CSHA, Canadian Study of Health and Aging; LMS, left main stem disease; LVEF, left ventricular ejection fraction; MI, myocardial infarction; NYHA, New York Heart Association Functional Classification; PA, pulmonary artery; PCI, percutaneous coronary intervention; TAVI, transcatheter aortic valve implantation.

### Model development

Between January 2009 and December 2014, up to 326 patients died within 30 days of the procedure (5.14%), with this decreasing to 4.14% between January 2013 and December 2014. In univariable analysis, patients with higher (mean-centred) body mass index (BMI), higher glomerular filtration rate and indication of sinus rhythm had decreased odds of 30-day mortality (table 2). Renal failure, extracardiac arteriopathy, calcification of ascending aorta, prior balloon aortic valvuloplasty, critical preoperative status, New York Heart Association class IV, poor mobility, CSHA frailty, KATZ (per point drop from 6 points), left ventricular ejection fraction <50%, non-elective procedure and non-transfemoral access each had increased odds of 30-day mortality.

**Table 2:** Univariable odds ratios (ORs) of each baseline variable on 30-day mortality in the main analysis development cohort

Variable*	OR (95% CI)†	P value
Mean-centred age	1.01 (1.00 to 1.03)	0.0751
Female	1.15 (0.92 to 1.44)	0.2111
Non-Caucasian	0.75 (0.40 to 1.43)	0.3840
Diabetic	0.86 (0.65 to 1.13)	0.2712
Current or ex-smoker	1.00 (0.80 to 1.25)	0.9893
Mean-centred BMI	0.97 (0.95 to 0.99)	0.0051
Glomerular filtration rate per 5 units increase	0.95 (0.92 to 0.97)	<0.0001
Renal failure	1.70 (1.16 to 2.49)	0.0062
Recent MI	1.65 (0.65 to 4.14)	0.2899
Pulmonary disease	1.27 (1.00 to 1.61)	0.0535
Cerebrovascular disease	0.92 (0.66 to 1.27)	0.6155
Extracardiac arteriopathy	1.61 (1.26 to 2.05)	<0.0001
Calcified aorta	1.34 (1.02 to 1.76)	0.0369
Sinus rhythm	0.76 (0.60 to 0.95)	0.0163
Previous cardiac surgery	0.88 (0.69 to 1.13)	0.3194
Prior BAV	1.46 (1.07 to 2.00)	0.0181
Previous PCI	0.89 (0.67 to 1.18)	0.4205
Critical preoperative state	2.94 (1.77 to 4.88)	<0.0001
NYHA class IV	1.49 (1.15 to 1.95)	0.0030
Poor mobility‡	2.74 (1.89 to 3.96)	<0.0001
CSHA frail‡	1.91 (1.33 to 2.75)	<0.0001
KATZ (per point drop from 6 points)‡	1.40 (1.24 to 1.58)	<0.0001
PA systolic >60 mm Hg	1.29 (0.96 to 1.75)	0.0946
Aortic peak gradient	1.00 (0.99 to 1.00)	0.0990
Aortic valve area per 0.1 unit increase	1.00 (0.95 to 1.04)	0.8450
LVEF<50%	1.33 (1.06 to 1.66)	0.0140
More than one diseased vessel	1.16 (0.93 to 1.46)	0.1832
Left main stem disease	1.00 (0.58 to 1.74)	0.9861
Non-elective procedure	1.77 (1.33 to 2.35)	<0.0001
Non-transfemoral access	2.12 (1.68 to 2.66)	<0.0001

*Variable definitions are given in online supplementary table 1.

†Each univariable OR was pooled across all 10 multiple imputed data sets.

‡The univariable ORs for the three frailty variables were estimated using only 2013–2014 data since this is the period in which these variables were recorded within the UK-TAVI Registry.

BAV, balloon aortic valvuloplasty; BMI, body mass index; CSHA, Canadian Study of Health and Aging; LVEF, left ventricular ejection fraction; MI, myocardial Infarction; NYHA, New York Heart Association Functional Classification; PA, pulmonary artery; PCI, percutaneous coronary intervention.

**Figure 3:**
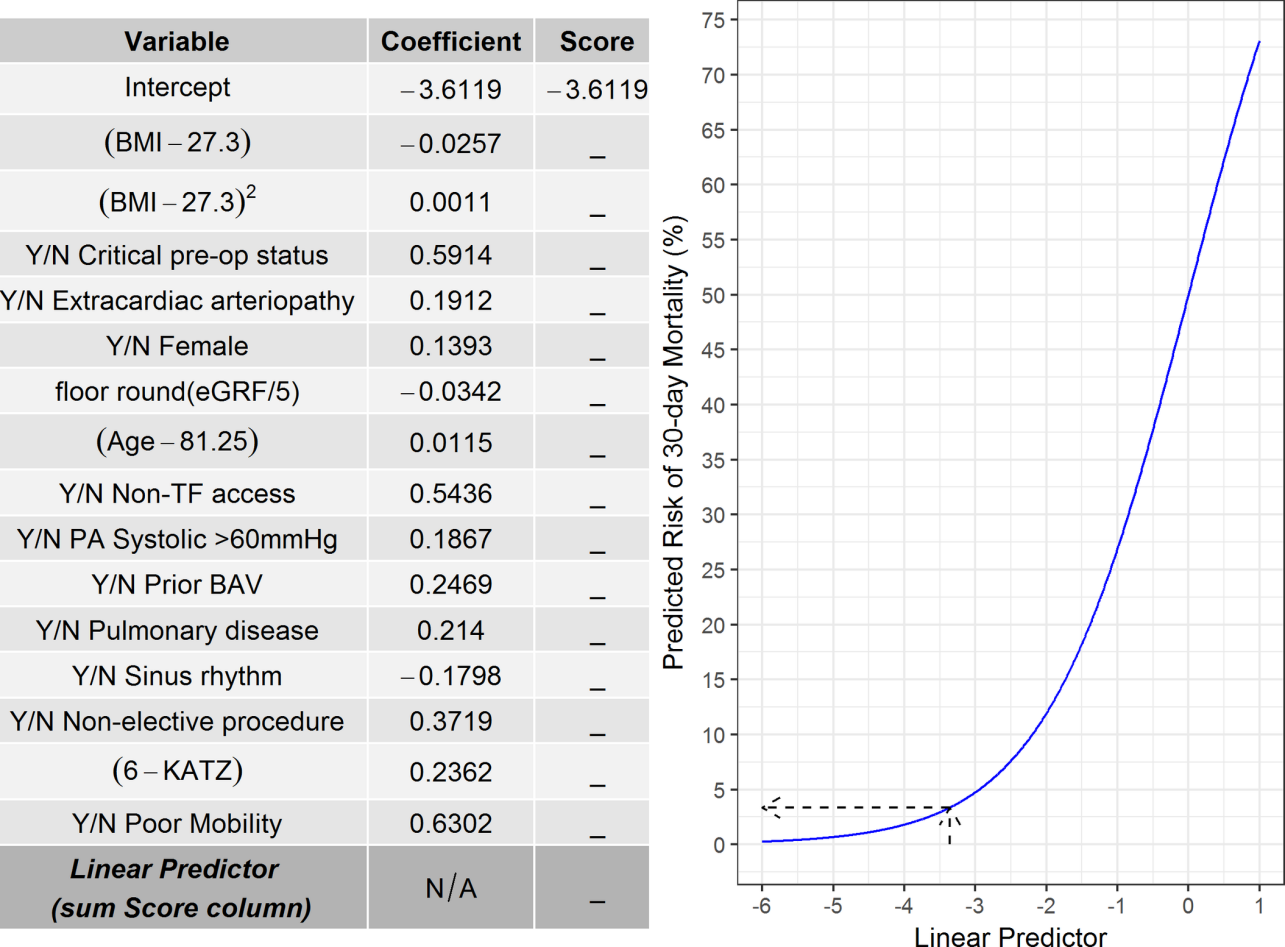
Graphical representation of the UK-TAVI (transcatheter aortic valve implantation) clinical prediction model (CPM). First, multiply each variable (either yes/no for categorical variables or enter the observed continuous variable) by the corresponding coefficient and then sum across all variables to obtain the linear predictor. The linear predictor can then be converted to a predicted risk using the graph or through the equation: exp(Linear Predictor)/{1+exp(Linear Predictor)}. The dotted arrows show the example described in the text. BAV, balloon aortic valvuloplasty; BMI, body mass index; eGFR, estimated glomerular filtration rate; N/A, not applicable; PA, pulmonary artery; TF, transfemoral access.

The variables selected in the final multivariable model (denoted UK-TAVI CPM) are given in table 3. The model intercept was −3.6119, with 15 variables included in the model, 2 of which were frailty measures (ie, KATZ and poor mobility). One can calculate the log odds of 30-day mortality for a new patient by multiplying each covariate by the corresponding coefficient in table 3 and summing across all variables—the so-called LP; the probability of 30-day mortality can then be obtained by transforming the LP as exp(LP)/{1+exp(LP)}. Figure 3 depicts a graphical representation of this process. For example, an 85-year-old woman with a BMI of 30 kg/m^2^, a glomerular filtration rate of 51 mL/min/1.73 m^2^ and KATZ score of 4 points would have log odds=−3.6119+((85−81.25)×0.0115)+(0.1393)+((30−27.3)×−0.0257)+((30−27.3)^2^×0.0011)+([51/5] ×−0.0342)+((6−4)×0.2362)=−3.3604, where [51/5]=10 (ie, floor rounding). This can be converted to a probability of 30-day mortality by exp(−3.3604)/{1+exp(−3.3604)}=0.0336×100=3.36% (dotted arrows in figure 3).

**Table 3:** Variables and coefficients included in the final multivariable UK-TAVI CPM

Variable*	Coefficient (SE)	OR (95% CI)
Intercept	−3.6119 (0.1995)	NA
Mean-centred age	0.0115 (0.0085)	1.012 (0.995 to 1.028)
Female	0.1393 (0.1174)	1.150 (0.913 to 1.447)
Mean-centred BMI	−0.0257 (0.0119)	0.975 (0.952 to 0.998)
Mean-centred BMI squared	0.0011 (0.0007)	1.001 (1.000 to 1.002)
Glomerular filtration rate per 5 units increase	−0.0342 (0.0139)	0.966 (0.940 to 0.993)
Pulmonary disease	0.2140 (0.1266)	1.239 (0.966 to 1.588)
Extracardiac arteriopathy	0.1912 (0.1348)	1.211 (0.930 to 1.577)
Sinus preoperative heart rhythm	−0.1798 (0.1193)	0.835 (0.661 to 1.056)
Prior BAV	0.2469 (0.1633)	1.280 (0.930 to 1.763)
Critical preoperative status	0.5914 (0.2770)	1.807 (1.050 to 3.109)
Poor mobility	0.6302 (0.2052)	1.878 (1.256 to 2.808)
KATZ (per point drop from 6 points)	0.2362 (0.0689)	1.267 (1.107 to 1.450)
PA systolic pressure >60 mm Hg	0.1867 (0.1583)	1.205 (0.884 to 1.644)
Non-elective procedure	0.3719 (0.1554)	1.451 (1.070 to 1.967)
Non-transfemoral access	0.5436 (0.1268)	1.722 (1.343 to 2.208)

*Variable definitions are given in online supplementary table 1.

BAV, balloon aortic valvuloplasty; BMI, body mass index; CPM, clinical prediction model; NA, not applicable; PA, pulmonary artery; TAVI, transcatheter aortic valve implantation.

### Model validation

The UK-TAVI CPM was well calibrated before and after bootstrap correction, with a calibration intercept and slope significantly close to 0 and 1, respectively (table 4); the apparent performance calibration plot is given in online supplementary figure 1. The apparent AUC of the model was 0.70 (95% CI 0.65 to 0.75), which reduced to 0.66 (95% CI 0.61 to 0.71) after bootstrap internal validation. The UK-TAVI CPM was well calibrated and had moderate discrimination across quantiles of observed predicted risk, although performance was marginally worse in the second and third quantiles (online supplementary table 2). Bootstrap-corrected performance across patient subgroups is given in online supplementary table 3.

**Table 4:** Performance measures before (apparent) and after bootstrap-corrected optimism within the 2013–2014 data (n=2969)

Validation	Calibration intercept (95% CI)	Calibration slope (95% CI)	AUC (95% CI)
Apparent	0.00 (−0.18 to 0.18)	1.00 (0.76 to 1.24)	0.70 (0.65 to 0.75)
Internal*	0.02 (−0.17 to 0.20)	0.79 (0.55 to 1.03)	0.66 (0.61 to 0.71)

*Estimated as the apparent performance minus optimism, where optimism was obtained through bootstrap resampling.

AUC, area under the curve.

**Figure 4:**
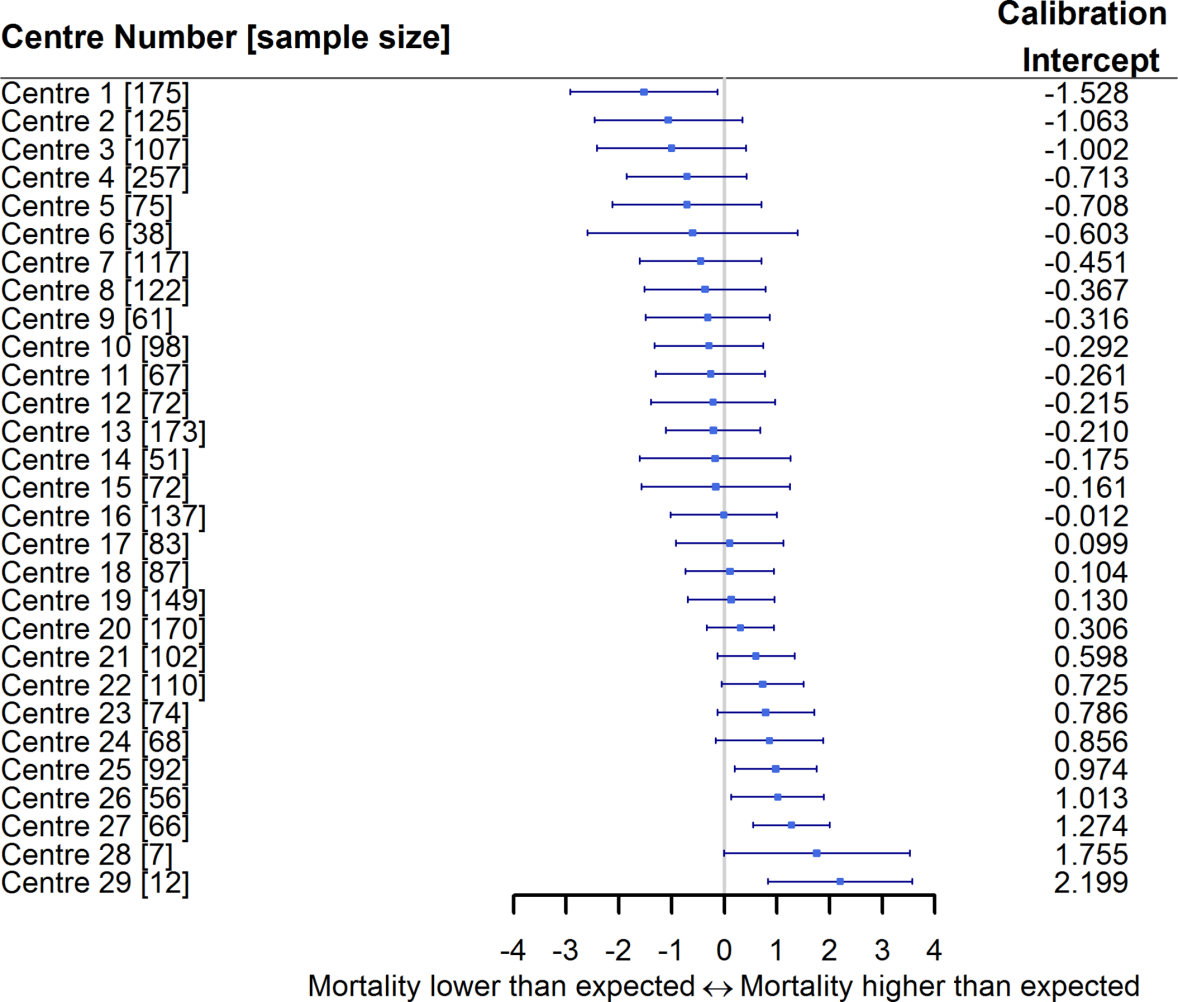
Forest plot of the calibration intercept for the UK-TAVI (transcatheter aortic valve implantation) clinical prediction model (CPM) across all centres. Centres have been sorted based on the calibration intercept. Three centres with no deaths by 30 days have been removed.

We examined the predictive performance across each centre, which demonstrated that the majority of centres had an observed event rate similar to that expected from the model (figure 4). However, there was heterogeneity in the calibration-in-the-large, with some centres performing ‘better’ than expected (calibration intercept <0), and others having an observed event rate higher than that expected by the model (calibration intercept >0).

### Sensitivity analyses

The first sensitivity analysis examined an alternative way of handling missing data, through a single imputation strategy. Most variables within the model derived under this approach were similar to those within the UK-TAVI CPM, but additionally included an indicator of calcified aorta, and aortic valve peak gradient (online supplementary table 4). The performance results were quantitatively similar to the main analysis model.

The second sensitivity analysis restricted the development cohort to those patients between January 2013 and December 2014 to contrast with the aforementioned two-stage modelling strategy (figure 1). The model developed under this approach included similar but fewer variables to the main analysis model (online supplementary table 5). After bootstrap internal validation, the calibration slope for 2013–2014 derived model was significantly below 1 (0.69, 95% CI 0.45 to 0.92), indicating overfitting within the smaller sample size (demonstrating an advantage of the two-stage modelling strategy). The internal validation AUC of the 2013–2014 derived model was 0.64 (95% CI 0.59 to 0.69).

## Discussion

This study has derived a TAVI risk prediction model for benchmarking and audit analyses in UK patients. After covariate selection, the UK-TAVI CPM included 15 variables, including 2 frailty-related measures. The model demonstrated strong calibration after correction for in-sample optimism, but only moderate discrimination. It is, therefore, of greater value in allowing comparison of risk-adjusted outcomes between centres than in aiding clinicians to provide accurate risk assessment to patients being offered TAVI.

Risk prediction in TAVI patients is an ongoing research area; the FRANCE-2,^9^ OBSERVANT,^10^ ACC^11^ and the herein UK-TAVI CPM each represent models derived exclusively on patients treated by TAVI and each share similar (clinically meaningful) risk factors. However, differences in the variables that are recorded across the national registries limit the ability to obtain a single generalisable model for practical use across countries. Thus, we have demonstrated the UK-TAVI CPM to be well calibrated both before and after correction for in-sample optimism, with an AUC of 0.70 and 0.66 within development and internal validation, respectively. Similar performance has been reported from the existing TAVI models within data sets similar to that in which they were developed.^9–11^ For example, the FRANCE-2 model had an AUC of 0.59 in a ‘test’ subset of the French registry,^9^ and the AUC within an internal validation of the ACC TAVI model was 0.66.^11^ However, a previous analysis of the UK registry found that the AUC of the existing TAVI CPMs was below 0.64 within the UK cohort.^13^

The limited discrimination of the UK-TAVI CPM means that this model is not yet ready for deployment when attempting to assess risk in individual cases. While one could question the need to use TAVI CPMs in such a capacity given the emerging evidence supporting expansion into low/intermediate-risk patients,^3^ CPMs are fundamental to appropriately adjust for case mix when investigating postprocedural outcomes across centres. In particular, the assessment of TAVI futility should consider multiple outcomes, including mortality, quality of life and hospital readmission, but the variability in the calibration intercept per TAVI centre (figure 4) indicates the importance of case-mix adjustment when comparing mortality rates. Consequently, the herein derived model presents an important step in facilitating appropriate adjustment for case mix when making centre-level outcome comparisons in UK patients, with similar applications undertaken within the STS/ACC Transcatheter Valve Therapy Registry using the ACC model.^11^

However, it is inevitable that predictive performance will decrease when the UK-TAVI CPM is applied in populations distinct to that in which it was developed (external validation),^22^ as observed when our group applied the France, Italian and American TAVI CPMs to the UK population.^13^ Since the primary aim of the UK-TAVI CPM is for national benchmarking, the main implementation of this model will be within UK patients. To this end, the split sample method of internal validation (randomly splitting the data into training and test sets) is common, but this is an inefficient use of data.^25^ Consequently, we applied bootstrapping methods to estimate the likely performance of the model in samples drawn from the registry. While lack of new and independent data from the UK registry meant that external validation was not possible, such an assessment of model performance will need to be undertaken by independent investigators.^29^ As such, we encourage the validation of our model in future extracts of the UK registry to assess temporal validation. Unquestionably, the UK-TAVI CPM should not be regarded as a static tool, and the discovery of novel predictors will be fundamental for deriving future TAVI models. The rapid development of TAVI techniques, knowledge and technology, combined with the possible expansion into lower risk patients, means the model should be continuously updated. The model needs to be maintained using contemporary extracts of the registry to avoid the calibration drift commonly observed in CPMs, such as that found in the logistic EuroSCORE.^30^ This would be equally applicable to other contemporary risk scores used elsewhere.

### Limitations

The main strength of this study is that TAVI data collection is mandatory in the UK, meaning the UK-TAVI CPM has been developed using comprehensive national data. However, several limitations need to be considered. First, since we excluded all patients from Northern Ireland and Scotland due to lack of independent mortality information, the model might not be representative of such patients. Second, this model only predicts 30-day mortality, but demonstrates the need for registries to routinely collect more wide-ranging outcomes (eg, hospital readmission, quality of life). Third, to maintain sufficient sample size, we included all data from 2009 to 2014, involving several iterations of valve types/devices. Although the modelling strategy aimed to revise the model using the 2013–2014 data, the UK-TAVI CPM will need to be updated in newer extracts to include patients exposed to only the most contemporary technology/practice. Finally, the generalisability of the model is unknown since we were unable to test the performance in independent data. Thus, we recommend independent researchers conduct future validation studies on the later extracts of the UK-TAVI Registry.

## Conclusions

This analysis of the UK-TAVI Registry has developed a contemporary risk model on over 6000 TAVI patients. The validation procedure demonstrated that the model was well calibrated but achieved limited discrimination. Thus, the derived model has potential to be used for benchmarking analyses in UK-TAVI patients, but is not yet ready for deployment when attempting to assess risk in individual cases. Future external validation studies of the model within the UK population are required, and outcomes other than 30-day mortality might usefully be explored.

**Key messages**

**What is already known on this subject?**

Existing clinical prediction models (CPM) for mortality following transcatheter aortic valve implantation (TAVI) have only moderate predictive performance when applied to populations distinct from those in which they were developed. Additionally, frailty has been shown to be an important predictor of mortality following TAVI, with the recent recording of frailty in national registries presenting opportunities to include such measures within the risk prediction.

**What might this study add?**

This study presents a contemporary multivariable CPM for predicting 30-day mortality after TAVI that included 15 risk factors, 2 of which were measures of frailty. This is the first model to be derived from the UK-TAVI Registry and presents an important step in facilitating appropriate adjustment for case mix when making centre-level outcome comparisons. The validation procedure demonstrated that the model was well calibrated and moderately discriminating.

**How might this impact on clinical practice?**

The model has potential to be used for benchmarking analyses in UK-TAVI patients by adjusting for case mix, and could facilitate clinical discussions around risk during the consent process. However, further development is needed as more data become available, and other outcomes should be explored.

## References

[1] Leon MB, Smith CR, Mack M, et al Transcatheter aortic-valve implantation for aortic stenosis in patients who cannot undergo surgery. N Engl J Med 2010;363:1597–607. 10.1056/NEJMoa1008232 20961243

[2] Smith CR, Leon MB, Mack MJ, et al Transcatheter versus surgical aortic-valve replacement in high-risk patients. N Engl J Med 2011;364:2187–98. 10.1056/NEJMoa1103510 21639811

[3] Leon MB, Smith CR, Mack MJ, et al Transcatheter or surgical aortic-valve replacement in intermediate-risk patients. N Engl J Med 2016;374:1609–20. 10.1056/NEJMoa1514616 27040324

[4] Vahanian A, Alfieri O, Andreotti F, et al Guidelines on the management of valvular heart disease (version 2012). Eur Heart J 2012;33:2451–96. 10.1093/eurheartj/ehs109 22922415

[5] Nashef SA, Roques F, Sharples LD, et al EuroSCORE II. Eur J Cardiothorac Surg 2012;41:734–45. 10.1093/ejcts/ezs043 22378855

[6] O’Brien SM, Shahian DM, Filardo G, et al The Society of thoracic surgeons 2008 cardiac surgery risk models: part 2-isolated valve surgery. Ann Thorac Surg 2009;88:S23–S42. 10.1016/j.athoracsur.2009.05.056 19559823

[7] Durand E, Borz B, Godin M, et al Performance analysis of EuroSCORE II compared to the original logistic EuroSCORE and STS scores for predicting 30-day mortality after transcatheter aortic valve replacement. Am J Cardiol 2013;111:891–7. 10.1016/j.amjcard.2012.11.056 23337835

[8] Ben-Dor I, Gaglia MA, Barbash IM, et al Comparison between society of thoracic surgeons score and logistic euroscore for predicting mortality in patients referred for transcatheter aortic valve implantation. Cardiovasc Revasc Med 2011;12:345–9. 10.1016/j.carrev.2011.04.005 21741324

[9] Iung B, Laouénan C, Himbert D, et al Predictive factors of early mortality after transcatheter aortic valve implantation: individual risk assessment using a simple score. Heart 2014;100:1016–23. 10.1136/heartjnl-2013-305314 24740804

[10] Capodanno D, Barbanti M, Tamburino C, et al A simple risk tool (the OBSERVANT score) for prediction of 30-day mortality after transcatheter aortic valve replacement. Am J Cardiol 2014;113:1851–8. 10.1016/j.amjcard.2014.03.014 24837264

[11] Edwards FH, Cohen DJ, O’Brien SM, et al Development and validation of a risk prediction model for in-hospital mortality after transcatheter aortic valve replacement. JAMA Cardiol 2016;1:46 10.1001/jamacardio.2015.0326 27437653

[12] Halkin A, Steinvil A, Witberg G, et al Mortality prediction following transcatheter aortic valve replacement: a quantitative comparison of risk scores derived from populations treated with either surgical or percutaneous aortic valve replacement. The Israeli TAVR Registry Risk Model Accuracy Assessment (IRRMA) study. Int J Cardiol 2016;215:227–31. 10.1016/j.ijcard.2016.04.038 27128536

[13] Martin GP, Sperrin M, Ludman PF, et al Inadequacy of existing clinical prediction models for predicting mortality after transcatheter aortic valve implantation. Am Heart J 2017;184:97–105. 10.1016/j.ahj.2016.10.020 28224933 PMC5333927

[14] Collins GS, Reitsma JB, Altman DG, et al Transparent reporting of a multivariable prediction model for individual prognosis or diagnosis (TRIPOD): the TRIPOD statement. BMJ 2015;350:g7594 10.1136/bmj.g7594 25569120

[15] Ludman PF The UK transcatheter aortic valve implantation registry; one of the suite of registries hosted by the National Institute for Cardiovascular Outcomes Research (NICOR). Heart 2012;98:1787–9. 10.1136/heartjnl-2012-302534 22879533

[16] Katz S, Ford AB, Moskowitz RW, et al Studies of illness in the aged. The index of adl: a standardized measure of biological and psychosocial function. JAMA 1963;185:914. 14044222 10.1001/jama.1963.03060120024016

[17] Rockwood K, Song X, MacKnight C, et al A global clinical measure of fitness and frailty in elderly people. CMAJ 2005;173:489–95. 10.1503/cmaj.050051 16129869 PMC1188185

[18] Rubin DB Multiple imputation for nonresponse in surveys: John Wiley & Sons, 1987.

[19] White IR, Royston P Imputing missing covariate values for the cox model. Stat Med 2009;28:1982–98. 10.1002/sim.3618 19452569 PMC2998703

[20] Vergouwe Y, Royston P, Moons KG, et al Development and validation of a prediction model with missing predictor data: a practical approach. J Clin Epidemiol 2010;63:205–14. 10.1016/j.jclinepi.2009.03.017 19596181

[21] Janssen KJ, Moons KG, Kalkman CJ, et al Updating methods improved the performance of a clinical prediction model in new patients. J Clin Epidemiol 2008;61:76–86. 10.1016/j.jclinepi.2007.04.018 18083464

[22] Steyerberg EW Clinical prediction models. New York: Springer, 2009.

[23] Shimura T, Yamamoto M, Kano S, et al Impact of the clinical frailty scale on outcomes after transcatheter aortic valve replacement. Circulation 2017;135:2013–24. 10.1161/CIRCULATIONAHA.116.025630 28302751

[24] Cox DR Two further applications of a model for binary regression. Biometrika 1958;45:562–5. 10.1093/biomet/45.3-4.562

[25] Austin PC, Steyerberg EW Events per variable (EPV) and the relative performance of different strategies for estimating the out-of-sample validity of logistic regression models. Stat Methods Med Res 2017;26:796–808. 10.1177/0962280214558972 25411322 PMC5394463

[26] R Core Team R. R: a language and environment for statistical computing. R Found. Stat. Comput 2017.

[27] Van BS, Groothuis-Oudshoorn K Mice: multivariate imputation by chained equations in R. J Stat Softw 2011;45:1–67.

[28] Robin X, Turck N, Hainard A, et al pROC: an open-source package for R and S+ to analyze and compare ROC curves. BMC Bioinformatics 2011;12:77 10.1186/1471-2105-12-77 21414208 PMC3068975

[29] Collins GS, Ma J, Gerry S, et al Risk prediction models in perioperative medicine: methodological considerations. Curr Anesthesiol Rep 2016;6:267–75. 10.1007/s40140-016-0171-8

[30] Hickey GL, Grant SW, Murphy GJ, et al Dynamic trends in cardiac surgery: why the logistic euroscore is no longer suitable for contemporary cardiac surgery and implications for future risk models. Eur J Cardiothorac Surg 2013;43:1146–52. 10.1093/ejcts/ezs584 23152436 PMC3655624

