# Supplementary material for: Novel United Kingdom prognostic model for 30-day mortality following transcatheter aortic valve implantation
Source: Heart. 2017 Dec 7;104(13):1109–16. doi: 10.1136/heartjnl-2017-312489 (PMC6031259; doi:10.1136/heartjnl-2017-312489)
Supplement: Supplementary file 1 [file heartjnl-2017-312489supp001.pdf]

Supplementary Material

A Novel UK Prognostic Model for 30-day Mortality following Transcatheter Aortic Valve Implantation

Glen P. Martin; Matthew Sperrin; Peter F. Ludman; Mark A. de Belder; Simon Redwood; John Townend; Mark Gunning; Neil E. Moat; Iain Buchan; Mamas A. Mamas

Supplementary Methods

Mathematical Details of Model Updating Techniques

We first derived a logistic regression CPM within the 2009-2014 data, considering only those variables that were observed throughout this period (i.e. not frailty related measures), by following the "majority method" of developing CPMs within multiple imputed data [1]. If P denotes the number of predictors that were selected using the majority method, then one can write the resulting linear predictor (LP) as

$$\text{LP}_i = \beta_0 + \sum_{p=1}^{P} \beta_p x_{i,p} \qquad (1)$$

for patient i, covariates $x_{i,p}$ for $p \in [1, P]$, and associated estimated coefficients β_p.

To ensure the model was reflective of most contemporary practice and to consider the addition of the three frailty measures, we performed model updating on equation (1). Such techniques, as previously described [2–5], have a hierarchical structure to alter a previously fitted CPM, to include potentially new variables. Specifically, we updated equation (1) by fitting a logistic regression model in the 2013-2014 UK TAVI registry with the LP (eq. 1), and the three frailty measures as covariates. Namely, we modelled

$$\log\left(\frac{\pi_i}{1-\pi_i}\right) = \hat{\alpha}_0 + \hat{\alpha}_1 \text{LP}_i + \delta_{CSHA} x_{i,\text{CSHA}} + \delta_{poor\ mobility} x_{i,\text{poor mobility}} + \delta_{KATZ} x_{i,\text{KATZ}}$$

where π_i denotes the predicted event probability for patient i in the 2013-14 UK TAVI registry, and $\boldsymbol{\delta}$ is the vector of parameters corresponding to each frailty covariate $\boldsymbol{x}$. The parameters $\hat{\alpha}_0$ and $\hat{\alpha}_1$ are the calibration intercept and slope, respectively. The likelihood ratio test was used to determine if any of the three frailty measures significantly improved the fit of the model, with only those meeting this criterion included in the final model; the null model included $\hat{\alpha}_0$ and $\hat{\alpha}_1$ (i.e. we at least applied model recalibration).

Bootstrapping for in-sample optimism correction

Bootstrap resampling was used to correct the predictive performance of the UK-TAVI CPM for in-sample optimism; the following steps were undertaken:

1. Extract the same random samples (obtained with replacement) from each imputed dataset.
2. Within each sampled imputed dataset, perform exactly the same model selection steps as described in the main paper (i.e. Figure 1). Pool the model coefficients across the bootstrap sampled imputed datasets – call this model the "bootstrap model".
3. Using the bootstrap model, calculate the "bootstrap apparent performance" within each bootstrap sampled imputed dataset and pool the results using Rubin's rules.
4. Apply the bootstrap model to the original imputed UK TAVI registry and calculate the performance (pooled across imputations) – call this the "out-of-sample performance".
5. Calculate the optimism as the difference between the bootstrap apparent performance and the out-of-sample performance.
6. Repeat steps 1-5 another 99 times.
7. Take the mean of the optimism estimates – call this mean the "in-sample optimism".

The optimism-corrected performance reported within the main paper is then the apparent performance estimates minus the in-sample optimism.

Supplementary Figures and Tables

Supplementary Figure 1: Calibration plot for the final UK-TAVI CPM. The blue line is a smoother through the observed points and the dashed black line indicates the null hypothesis that the model is perfectly calibrated.

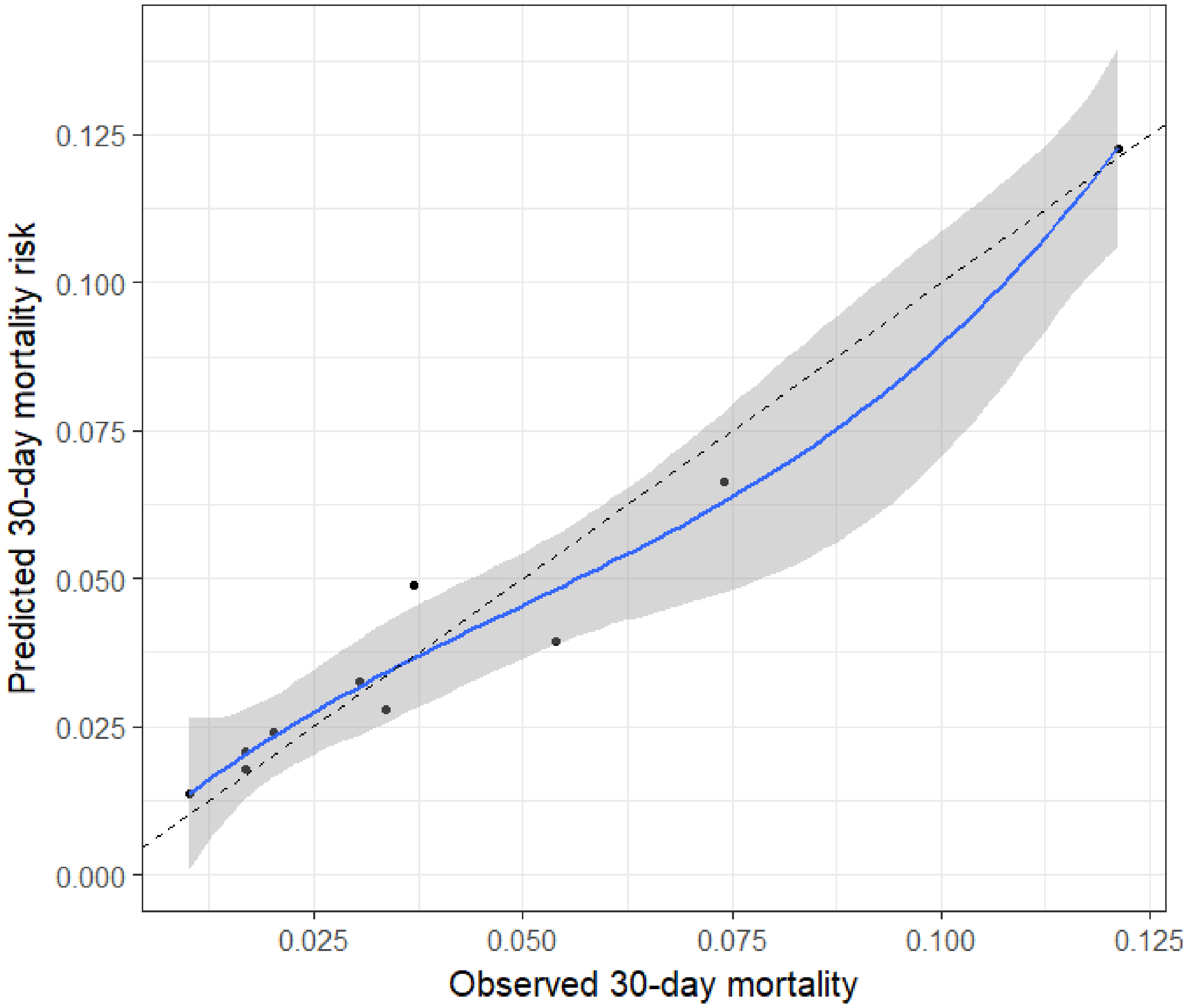

Supplementary Table 1: Variable definitions and coding in the UK TAVI registry of all candidate risk factors.

**Variable**	**Definition**	**UK TAVI Registry Variable**
Mean-centred Age (squared and cubed terms also considered)	Age of the patient at time of procedure minus 81.25 years	Age at Op
Female	Female sex	1.07 Sex
Non-Caucasian	Any ethnic origin other than white	1.08 Ethnic origin
Diabetic	Diabetic patient with any of the following treatments: dietary control, insulin, oral medication	3.01 Diabetes
Smoker	Current or ex-smoker	3.02 Smoking status
Mean-centred BMI (squared terms also considered)	The BMI of the patient minus 27.3	5.02 Weight and 5.01 Height
Glomerular Filtration Rate per 5 unit increase	Calculated by the Modification of Diet in Renal Disease formula, per 5 unit increase *	Age at Op, 1.07.Sex, 1.08.Ethnic.origin and 3.03 Creatinine
Renal Failure	Patient is on dialysis or has a creatinine>200 μmol/L	3.03 Creatinine and 3.041 On dialysis
Recent MI	Any previous MI within 30 days prior to the date of the TAVI operation	3.05 Previous MI and interval between procedure and last MI
Pulmonary disease	Any of the following: (1) Asthma, or (2) COAD /emphysema, or (3) Other significant pulmonary disease	3.06 History of pulmonary disease
Cerebrovascular Disease	Any of the following: (1) CVA with full recovery, or (2) CVA with residual deficit, or (3) TIA or RIND	3.08 History of neurological disease
Extracardiac arteriopathy	Patient has extracardiac arteriopathy	3.09 Extracardiac arteriopathy
Calcified Aorta	Patient has extensive calcification of ascending aorta (grade 3 or 4)	3.10 Extensive calcification of ascending aorta
Sinus Rhythm	Patient has sinus pre-operative rhythm	3.11 Preoperative heart rhythm
Previous Cardiac operation	Any of the following: (1) Previous CABG, or (2) Previous valve operation, or (3) Other operation requiring opening or the pericardium	4.01 Previous cardiac surgery
Prior BAV	Any BAV performed prior (upstream) of the current	4.021 Balloon aortic valvuloplasty prior to

	TAVI procedure	date of TAVI
Previous PCI	Patient had a PCI prior to date of TAVI	4.03 Previous PCI
Critical pre-operative status	Any of the following options: (1) Ventricular tachycardia or fibrillation or aborted sudden death, or (2) Preoperative cardiac massage, or (3) Preoperative ventilation before anaesthetic room, or (4) Preoperative inotropes or IABP, or (5) Preoperative acute renal failure (anuria or oliguria <10ml/hr)	5.031 Critical preoperative status v4
NYHA class IV	NYHA dyspnoea status recorded as "4. Symptoms at rest or minimal activity"	5.05 NYHA dyspnoea status (Pre-procedure; stable only)
Poor mobility	Poor mobility (Severe impairment of mobility secondary to musculoskeletal or neurological dysfunction)	3.091 Poor mobility
CSHA	CSHA Clinical Frailty Scale score with any option **except**: (1) very fit, or (2) well, or (3) apparently vulnerable	5.051 CSHA Clinical Frailty Scale score
KATZ (per point drop from 6 points)	Katz Index of Independence in Activities of Daily Living, number of points less than 6 (e.g. observed KATZ of 2 points becomes 4 after the transformation)	6 points - 5.052 Katz Index of Independence in Activities of Daily Living
PA Systolic pressure >60mmHg	PA systolic > 60mmHg	6.012 PA systolic pressure (mmHg)
Aortic peak gradient	Aortic valve peak gradient (mmHg)	6.02 Aortic valve peak gradient
Aortic valve area per 0.1 unit increase	Aortic valve area for every 0.1 cm^2 increase	6.03 Aortic valve area
LVEF <50%	Left ventricular ejection fraction less than 50%	6.08 LV function
More than one diseased vessel	Extent of coronary vessel disease (ignoring LMS disease) with any option **other than** "no vessel with >50% diameter stenosis"	6.09 Extent of coronary vessel disease
Left main stem disease	Left main stem disease with >50% diameter stenosis	6.10 Left main stem disease
Non-elective procedure	Any procedure that was (1) urgent, or (2) emergency, or (3) salvage	7.06 Procedure urgency

Non-transfemoral access	Any access route **other than** Femoral (percutaneous) or Femoral (surgical)	7.10 Delivery approach

*: GFR (mL/min/1.73 m^2) = 175 x (Serum creatinine [mg/dL])$^{-1.154}$ x (Age)$^{-0.203}$ x (0.742 if female) x (1.212 if ethnic origin recorded as “other” in UK TAVI registry)

Supplementary Table 2: Apparent model performance across empirical quantiles of predicted risk from the UK-TAVI CPM.

Quartile (predicted risk range)	Calibration Intercept (95% CI)	Calibration Slope (95% CI)	AUC (95% CI)
1st (0% - 2.07%)	0.04 (-0.54, 0.62)	2.33 (-1.39, 6.05)	0.60 (0.45, 0.75)
2nd (2.07% - 2.89%)	-0.13 (-0.65, 0.39)	4.32 (-1.05, 9.69)	0.63 (0.50, 0.77)
3rd (2.98% - 4.84%)	0.18 (-0.20, 0.55)	0.90 (-1.65, 3.45)	0.54 (0.44, 0.64)
4th (4.84% - 44.4%)	-0.06 (-0.33, 0.21)	1.12 (0.60, 1.65)	0.66 (0.59, 0.74)

Supplementary Table 3: Bootstrap corrected model performance for the UK-TAVI CPM across different patient subgroups.

**Subgroup**	**Calibration Intercept (95% CI)**	**Calibration Slope (95% CI)**	**AUC (95% CI)**
Age			
<85 years	0.06 (-0.18, 0.29)	0.86 (0.57, 1.15)	0.68 (0.62, 0.74)
≥85 years	-0.04 (-0.34, 0.25)	0.64 (0.22, 1.06)	0.61 (0.53, 0.70)
Sex			
Male	0.06 (-0.20, 0.31)	0.92 (0.59, 1.24)	0.68 (0.61, 0.75)
Female	-0.03 (-0.29, 0.24)	0.65 (0.30, 1.01)	0.63 (0.56, 0.70)
Renal Failure			
Yes	0.01 (-0.64, 0.67)	0.82 (0.01, 1.64)	0.66 (0.46, 0.86)
No	0.02 (-0.17, 0.21)	0.78 (0.53, 1.04)	0.66 (0.61, 0.71)
Previous Cardiac Surgery			
Yes	0.18 (-0.15, 0.50)	0.63 (0.23, 1.02)	0.66 (0.57, 0.74)
No	-0.05 (-0.28, 0.17)	0.89 (0.59, 1.19)	0.66 (0.60, 0.72)
NYHA			
Class IV	0.19 (-0.18, 0.56)	0.78 (0.35, 1.21)	0.69 (0.60, 0.79)
Class I – III	-0.04 (-0.25, 0.18)	0.74 (0.44, 1.04)	0.64 (0.58, 0.69)
LVEF			
<50%	0.09 (-0.18, 0.37)	1.13 (0.77, 1.49)	0.75 (0.69, 0.81)
≥50%	-0.04 (-0.29, 0.21)	0.44 (0.09, 0.79)	0.58 (0.51, 0.65)
Frailty			
KATZ=6	-0.03 (-0.30, 0.24)	0.54 (0.04, 1.05)	0.57 (0.50, 0.65)
KATZ<6	0.06 (-0.20, 0.32)	0.85 (0.48, 1.22)	0.67 (0.61, 0.73)
Poor Mobility	0.02 (-0.27, 0.30)	0.76 (0.29, 1.22)	0.62 (0.54, 0.70)
Normal Mobility	0.02 (-0.22, 0.26)	0.81 (0.39, 1.24)	0.62 (0.56, 0.69)
Access route			
Transfemoral	0.03 (-0.19, 0.25)	0.90 (0.61, 1.19)	0.66 (0.60, 0.72)
Non-Transfemoral	-0.02 (-0.36, 0.32)	0.50 (-0.02, 1.01)	0.58 (0.48, 0.68)
Valve type			
SAPIEN	0.07 (-0.16, 0.30)	0.66 (0.36, 0.97)	0.64 (0.57, 0.70)
CoreValve	0.06 (-0.26, 0.37)	0.97 (0.57, 1.38)	0.69 (0.62, 0.77)

Supplementary Table 4: Variables included in the model within the sensitivity analysis that only singularly imputed missing data.

**Variable ***	**Coefficient (SE)**	**OR (95% CI)**
Intercept	-3.3855 (0.2432)	N/A
Mean-centred Age	0.0131 (0.0085)	1.0132 (0.9965, 1.0302)
Female	0.1605 (0.1185)	1.1741 (0.9308, 1.4810)
Mean-centred BMI	-0.0267 (0.0118)	0.9736 (0.9514, 0.9964)
Mean-centred BMI squared	0.0011 (0.0000)	1.0011 (0.9998, 1.0024)
Glomerular Filtration Rate per 5 unit increase	-0.0346 (0.014)	0.966 (0.9398, 0.9929)
Pulmonary disease	0.2103 (0.1253)	1.2340 (0.9653, 1.5776)
Extracardiac arteriopathy	0.1837 (0.1350)	1.2016 (0.9222, 1.5657)
Calcified Aorta	0.1785 (0.1430)	1.1955 (0.9032, 1.5823)
Sinus pre-operative heart rhythm	-0.1522 (0.1197)	0.8588 (0.6792, 1.0860)
Prior BAV	0.2742 (0.1636)	1.3155 (0.9547, 1.8128)
Critical pre-operative status	0.6772 (0.3129)	1.9684 (1.066, 3.6347)
Poor Mobility	0.6713 (0.2023)	1.9567 (1.3161, 2.9092)
KATZ (per point drop from 6 points)	0.2053 (0.0679)	1.2279 (1.0749, 1.4028)
PA Systolic pressure >60mmHg	0.2884 (0.1604)	1.3343 (0.9743, 1.8273)
Aortic peak gradient	-0.0032 (0.0023)	0.9968 (0.9923, 1.0014)
Non-elective procedure	0.3740 (0.1575)	1.4536 (1.0675, 1.9793)
Non-transfemoral access	0.5699 (0.1265)	1.7682 (1.3798, 2.2659)

Abbreviations: BAV: Balloon aortic valvuloplasty, BMI: Body mass index, OR: Odds ratio, SE: standard error

**: Variable definitions are given in Supplementary Table 1.*

Supplementary Table 5: Variables included in the model within the sensitivity analysis that limited the development cohort to those procedures between January 2013 and December 2014.

**Variable ***	**Coefficient (SE)**	**OR (95% CI)**
Intercept	-3.1635 (0.3166)	N/A
Mean-centred Age	0.0069 (0.0134)	1.0070 (0.9810, 1.0337)
Female	0.0292 (0.1904)	1.0297 (0.7090, 1.4953)
Diabetes	-0.4040 (0.2538)	0.6676 (0.4060, 1.0980)
Mean-centred BMI	-0.0294 (0.0184)	0.9710 (0.9367, 1.0066)
Glomerular Filtration Rate per 5 unit increase	-0.0492 (0.0221)	0.9520 (0.9116, 0.9941)
Pulmonary disease	0.3507 (0.1991)	1.4201 (0.9613, 2.0979)
Sinus pre-operative heart rhythm	-0.3001 (0.1932)	0.7407 (0.5072, 1.0817)
NYHA Class IV	0.4559 (0.2213)	1.5775 (1.0224, 2.4340)
Poor Mobility	0.6496 (0.2067)	1.9148 (1.2769, 2.8715)
KATZ (per point drop from 6 points)	0.2550 (0.0687)	1.2904 (1.1279, 1.4763)
Non-transfemoral access	0.5557 (0.2103)	1.7431 (1.1544, 2.6322)

Abbreviations: BAV: Balloon aortic valvuloplasty, BMI: Body mass index, NYHA: New York Heart Association Functional Classification, OR: Odds ratio, SE: standard error

**: Variable definitions are given in Supplementary Table 1.*

Supplementary References

1 Vergouwe Y, Royston P, Moons KGM, *et al.* Development and validation of a prediction model with missing predictor data: a practical approach. *J Clin Epidemiol* 2010;**63**:205–14. doi:10.1016/j.jclinepi.2009.03.017

2 Janssen KJM, Moons KGM, Kalkman CJ, *et al.* Updating methods improved the performance of a clinical prediction model in new patients. *J Clin Epidemiol* 2008;**61**:76–86. doi:10.1016/j.jclinepi.2007.04.018

3 Steyerberg EW, Borsboom GJJM, van Houwelingen HC, *et al.* Validation and updating of predictive logistic regression models: a study on sample size and shrinkage. *Stat Med* 2004;**23**:2567–86. doi:10.1002/sim.1844

4 Moons KGM, Kengne AP, Grobbee DE, *et al.* Risk prediction models: II. External validation, model updating, and impact assessment. *Heart* 2012;**98**:691–8. doi:10.1136/heartjnl-2011-301247

5 Su T-L, Jaki T, Hickey GL, *et al.* A review of statistical updating methods for clinical prediction models. *Stat Methods Med Res* Published Online First: 26 July 2016. doi:10.1177/0962280215626466
